# Understanding financial risk protection in China’s health system: a descriptive analysis using data from multiple national household surveys

**DOI:** 10.1186/s12889-023-16679-4

**Published:** 2023-09-19

**Authors:** Yuanyuan Li, Hongcai Guan, Hongqiao Fu

**Affiliations:** School of Public Health, Peking University Health Science Center, Beijing, China

**Keywords:** Financial risk protection, Catastrophic health expenditure, Medical impoverishment, Health system

## Abstract

**Background:**

Providing financial risk protection is one of the fundamental goals of health systems. Catastrophic health expenditure (CHE) and medical impoverishment (MI) are two common indicators in evaluating financial risk protection in health. As China continues its health system reform to provide accessible and affordable health care, it is important to have a clear understanding of China’s progress in financial risk protection. However, past research showed discrepancies in the incidence of CHE and MI. In this article, using data from four national household surveys, we analyzed levels and characteristics of CHE and MI in China under different definitions.

**Methods:**

We used multiple conventional thresholds for CHE and MI to comprehensively describe the levels of financial risk protection in China. We used data from four national household surveys to measure the incidence of CHE and MI, and their inequalities by urban/rural status and by income quartiles. The Probit regression model was used to explore influencing factors of CHE and MI.

**Results:**

We found that the incidences of CHE and MI were largely consistent across four national household surveys, despite different sampling methods and questionnaire designs. At the 40% nonfood expenditure threshold, the incidence of CHE in China was 14.95%-17.73% across four surveys during the period of 2016–2017. Meanwhile, at the 1.9 US dollars poverty line, the incidence of MI was 2.01%-5.63%. Moreover, rural residents, lower-income subgroups, and smaller households were faced with higher financial risks from healthcare expenditures. Although positive progress in financial risk protection has been achieved in recent years, China has disproportionately high incidences of CHE and MI, compared to other countries.

**Conclusion:**

China has large margins for improvements in risk financial protection, with large inequalities across subgroups. Providing better financial protection for low-income groups in rural areas is the key to improve financial protection in China.

**Supplementary Information:**

The online version contains supplementary material available at 10.1186/s12889-023-16679-4.

## Background

Providing financial risk protection is one of the fundamental goals of health systems [1]. It is a key dimension of universal health coverage (UHC) that is a core element of the Sustainable Development Goals (SDGs) [2]. Catastrophic Health Expenditure (CHE) and Medical Impoverishment (MI) are two commonly used indicators to assess financial risk protection in international reports [3] and international literature [4–7]. They both measure financial hardship caused by healthcare expenditures: the former emphasizes the relatively large size of healthcare expenditures and the latter captures the hardship’s implications in falling into poverty [6, 7]. Specifically, CHE means that a household’s out-of-pocket healthcare expenditure (excluding reimbursement by a third party) exceeds its capacity to pay [2, 6]. MI means that a household’s consumption expenditure is above the poverty line in total but lower than the poverty line after deducting the healthcare expenditure [2, 7]. World Health Organization (WHO) and World Bank (WB) set the goal that all countries should achieve 100% of financial risk protection by 2030 [8].

Since the severe acute respiratory syndrome (SARS) epidemic in 2003, China has devoted to reforming its health system to provide accessible and affordable healthcare services with reasonable quality for all citizens [9]. The Chinese government has successively established or expanded the New Cooperative Medical Scheme (NCMS) for rural residents, the Urban Employee Basic Medical Insurance (UEBMI) for employed urban residents, and the Urban Resident Basic Medical Insurance (URBMI) for child, elderly, disabled, and unemployed urban residents [10]. According to the China Statistical Yearbook, the number of people enrolled in the three health insurance schemes reached 1.361 billion in 2020, covering 96% of the population. Moreover, China has established the medical assistance program and Critical Illness Insurance scheme [11], aiming to improve financial protection for Chinese population. However, the incidences of CHE and MI remained largely unchanged in the past twenty years [10, 12].

Existing literature has extensively evaluated the levels of CHE and MI in China [12–14]. However, results from the literature were inconsistent, suggesting no consensus on the incidences of CHE and MI in China. On the one hand, differences in previous results were partly due to the different datasets they used. Surveys used to evaluate the incidences of CHE and MI differed in sampling methods, sample selection, and questionnaire designs. For instance, the number of items asked when collecting healthcare expenditures and the recall period of the questionnaire were found to have considerable impacts on the measurement of CHE [15]. On the other hand, various definitions were adopted when calculating the incidence of CHE and MI, which made findings from different studies incomparable [16]. For instance, household consumption, household consumption net of food consumption, and household income were ever used in the estimation of CHE. Therefore, it is necessary to use different surveys to compare the incidences of CHE and MI under the same calculation methods, which would help us better understand financial risk protection in China.

Our study aims to calculate the incidence of CHE and MI using different surveys and thresholds to better understand financial risk protection in China. Following the guidance of the Chinese National Bureau of Statistics and existing literature [9, 10], we also investigate heterogeneity by residency (urban/rural area) and income quartiles to capture disparities across socioeconomic groups. Moreover, we explore the influencing factors of CHE and MI. Our results would provide implications for China to accelerate the UHC progress in China through understanding the current status, identifying disadvantaged populations, and developing effective policy responses. As the largest developing country, financial risk protection in China would also provide a lesson for other developing countries.

## Methods

### Data sources

To describe the levels and characteristics of financial risk protection in China’s health system, we used four nationally representative household surveys, including China Family Panel Studies (CFPS), Chinese General Social Survey (CGSS), China Household Finance Survey (CHFS), and Chinese Social Survey (CSS). To ensure the incidences of CHE and MI from multiple surveys comparable in terms of the time frame, this study selected waves of 2016–2017 in these four household surveys for empirical analysis. Moreover, we used the CFPS data from 2010 to 2020 to conduct trend analysis to examine changes of CHE and MI over the past ten years. Details of the four surveys are described below.

#### CFPS

CFPS (http://www.isss.pku.edu.cn/cfps/) is a nationally representative survey organized by Peking University, aiming to capture the changes in population, society, economy, health, and education through longitudinal data collection. CFPS adopts a multi-stage probability sampling method, covering approximately 15,000 households in 25 provinces. The first wave of CFPS was conducted in 2010, and currently, there are six available waves, including data from 2010, 2012, 2014, 2016, 2018, and 2020. This survey has been extensively utilized to evaluate the performance of the health system in China, including financial risk protection [9, 13, 17, 18].

#### CGSS

CGSS (http://cgss.ruc.edu.cn/) is a multiyear cross-sectional survey organized by Renmin University of China, with the objective of describing the changes in social structure and quality of life in China. CGSS employs a multi-stage stratified sampling design to ensure being nationally representative. Since 2003, CGSS has conducted cross-sectional surveys involving over 10,000 households every one or two years. However, it should be noted that the sample size is around 3,000 households due to missing expenditure data in our analysis. CGSS has been used in research examining the determinants of health [19, 20].

#### CHFS

CHFS (https://chfs.swufe.edu.cn/) is led by Southwestern University of Finance and Economics, with the goal of gathering information on household finance at the micro level to describe the economic and financial behaviors of households. The survey adopts a random sampling method, and there are five waves currently available, including 2011, 2013, 2015, 2017, and 2019. CHFS 2017, the wave we used in this study, covered 40,011 households in 29 provinces. The samples collected from CHFS effectively represent households throughout the entire country. CHFS has been employed in various research topics, including assessing the health payment and the incidence of CHE and MI [21, 22].

#### CSS

CSS (http://csqr.cass.cn/) is carried out by the Institute of Sociology, Chinese Academy of Social Sciences, with the primary aim of describing the social changes occurring during China’s transition period. This goal is achieved through biennial longitudinal surveys with a focus on employment, family, and social attitudes. CSS employs a rigorous probability sampling method, covering 7,000 to 10,000 households across all 31 provinces in China. The sampled households can effectively mirror the diverse characteristics between the ages of 18 to 69 years old within the Chinese population. There are eight waves currently available, including 2006, 2008, 2011, 2013, 2015, 2017, 2019, and 2021. CSS has been used in health system performance evaluation and subjective feelings towards health issues among citizens [23, 24].

### Indicators and core variables

As shown in Table 1, we used multiple definitions with various thresholds for CHE and MI to comprehensively describe the levels of financial risk protection in China.

**Table 1:** Definitions of CHE and MI by different thresholds

Indicators	Detail definitions
CHE	
40% nonfood expenditure	The proportion of households whose healthcare expenditure were at least 40% of the household non-food expenditure
10% expenditure	The proportion of households whose healthcare expenditure were at least 10% of the household total consumption expenditure
25% expenditure	The proportion of households whose healthcare expenditure were at least 25% of the household total consumption expenditure
MI	
1.9 USD poverty line	The difference in the poverty headcount with and without out-of-pocket healthcare expenditure included in household total consumption. Poverty line is defined as 1.9 US dollars per person-day in 2011 and is adjusted by purchasing price parity and consumer price index
3.1 USD poverty line	The difference in the poverty headcount with and without out-of-pocket healthcare expenditure included in household total consumption. Poverty line is defined as 3.1 US dollars per person-day in 2011 and is adjusted by purchasing price parity and consumer price index
Relative poverty line	The difference in the poverty headcount with and without out-of-pocket healthcare expenditure included in household total consumption. Poverty line is defined as 60% of median per capita total consumption expenditure

*CHE* Catastrophic health expenditure, *MI* Medical impoverishment

In this study, we used three definitions of CHE, including 40% of household nonfood expenditure, 10% of household total consumption expenditure, and 25% of household total consumption expenditure. These three definitions were widely used in WHO reports and international literature [2, 3, 6, 9]. The 40% nonfood threshold was often used in early literature and reflected financial hardship caused by healthcare expenditure after subsistence needs were met [25]. The 10% and the 25% thresholds were often used to evaluate the progress in UHC targets. It should be noted that, in addition to CFPS 2016, CGSS 2017, CHFS 2017, and CSS 2017 to measure the incidence of CHE and MI during 2016–2017, we used CFPS 2010–2020 to examine changes in CHE and MI over the last ten years. Questionnaires on total household expenditure and household healthcare expenditures in the other three surveys were not consistent over years.

We used three thresholds of poverty line to estimate the incidence of MI, including 1.9 US dollars (USD) per day per person, 3.1 USD per day per person, and a relative poverty line that equals to 60% of median expenditure [3, 7]. The 1.9 USD poverty line was widely used to monitor the SDGs, while the 3.1 USD poverty line was more commonly used for middle-income countries and the relative poverty line was often used for OECD countries [7]. Since the poverty lines of 1.9 US dollars and 3.1 US dollars were in 2011 USD, we used Purchasing Power Parity (PPP) and Consumer Price Index (CPI) to convert the poverty lines to equivalent RMB values in 2017. After adjustment, 1.9 USD in 2011 was equivalent to 7.56 (1.9 × 3.52 × 1.13) RMB in 2017, and 3.1 USD in 2011 was equivalent to 12.33 (3.1 × 3.52 × 1.13) RMB in 2017. Therefore, the poverty lines of 1.9 USD and 3.1 USD per person per day were equivalent to 2757.46 and 4499.01 RMB per person-year in 2017, respectively.

Table 2 reports the variables used to estimate CHE and MI in each survey. As each survey collected data on household expenditures through different items and different wording of questions, it was important to examine variations in the definitions of these variables across four surveys. As shown in Table 2, the definitions of the variables were generally identical, with slight differences across the four surveys. When examining household food expenditure, CFPS 2016 collected the expenses of basic food, snacks, beverages, cigarettes, and alcohols. In contrast, CGSS 2017 and CSS 2017 focused on basic food, and the market value of home-grown farm products. Another example is the household healthcare expenditure. CSS 2017 solely collected household out-of-pocket healthcare expenditure, while CFPS 2016 and CHFS 2017 also accounted for costs associated with exercise and dietary supplements. Therefore, samples obtained from the four surveys were not entirely identical due to varied definitions, which could affect the measurement of CHE and MI. To comprehensively understand financial risk protection in China’s health system, it is crucial to compare the findings from different surveys.

**Table 2:** Detailed definitions of core variables from different household surveys

	CFPS 2016	CGSS 2017	CHFS 2017	CSS 2017
Household consumption expenditure	Household consumption expenditures last year, including expenditures on food, housing, health care, mortgage, and other consumptions	Household consumption expenditures last year, including expenditures on food, clothing, housing, durable goods, consumer goods, transportation and communication, culture and entertainment, education and training, health care, and online shopping	Household consumption expenditure last year, including expenditures on food, clothing, housing, household facilities articles and services, health care, transportation and communication, education and entertainment, and other consumptions	Household consumption expenditure last year, including expenditures on food, clothing, house rent, mortgage, electricity, water, and gas, property fee, heating fee, household appliances, furniture, and household vehicles spending, health care, transportation and communication, education, culture, entertainment and tourism
Household food expenditure	The average monthly household food expenditure last year, and snacks, beverages, cigarettes, and alcohols spending, including eating at home and eating out	Household food expenditure last year, including the market value of home-grown farm products	The average monthly household food expenditure last year, including expenditures on eating out, cigarettes, alcohols and beverages, and the market value of home-grown farm products	Household food expenditure last year, including eating out and the market value of home-grown farm products
Household healthcare expenditure	Household out-of-pocket healthcare expenditure last year, including exercising and dietary supplements	Household out-of-pocket healthcare expenditure last year	Household healthcare expenditure last year, and expenditures on exercising, including healthcare products, physical therapy, gym membership, fitness equipment and food, and personal trainer	Household out-of-pocket healthcare expenditure last year, including outpatient and inpatient expenditures
Household income	Household net income last year	Household total income last year	Household total income last year	Household total income last year

*CFPS* China Family Panel Studies, *CGSS* Chinese General Social Survey, *CHFS* China Household Finance Survey, *CSS* Chinese Social Survey

### Statistical analysis

To provide an overview of the similarities and differences between the four surveys, summary statistics were calculated for each dataset. Both unweighted and weighted statistics were calculated to ensure a comprehensive analysis. Descriptive statistics for the weighted incidence of CHE and MI were also calculated for each survey and each threshold. To maintain national representativeness, cross-sectional weights from each survey were applied. In addition, descriptive statistics for the incidence of CHE and MI were reported by urban/rural status and by income quartiles. Chi-square test was employed to test the differences between subgroups for statistical significance.

In exploring the factors influencing CHE and MI, a Probit regression model was utilized. The model was set as follows:

1 $${Y}_{i}=\alpha + {\delta }_{1} \times {Urban}_{i}+ {\delta }_{2} \times {IncomeGroup}_{i}+ {\delta }_{3} \times {Familysize}_{i}+ {\delta }_{4} \times {Exp\_capita}_{i} + {\delta }_{5} \times {Food\_capita}_{i}+{X}_{i}\beta + {\varepsilon }_{i}$$

The subscript $i$ represents a household. ${Y}_{i}$ is a dummy variable that indicates whether CHE or MI occurred. ${Urban}_{i}$ is a dummy variable that equals to 1 if a household is in the urban area and 0 if the household is in the rural area. ${IncomeGroup}_{i}$ is a vector of dummy variables that represents quartile subgroups by household income per capita. ${Familysize}_{i}$ represents household family size. ${Exp\_capita}_{i}$ represents household consumption expenditure per capita. ${Food\_capita}_{i}$ represents household food expenditure per capita. ${X}_{i}$ represents a vector of dummy variables representing each province.

## Results

### Summary statistics

Table 3 shows both unweighted and weighted summary statistics of CFPS 2016, CGSS 2017, CHFS 2017, and CSS 2017. In each dataset, urban households accounted for more than half of the sample. Before weighting, urban households comprised 51%, 63%, 68%, and 54% of the sample for CFPS 2016, CGSS 2017, CHFS 2017, and CSS 2017, respectively. When weighted, the proportion of urban households increased to 56%, 64%, 71%, and 58% in each survey, respectively.

**Table 3:** Summary statistics

	CFPS 2016	CGSS 2017	CHFS 2017	CSS 2017
	Mean (95% CI)	Mean (95% CI)	Mean (95% CI)	Mean (95% CI)
Unweighted				
Urban	0.51(0.50–0.51)	0.63(0.62–0.65)	0.68(0.68–0.69)	0.54(0.53–0.55)
Household family size (person per household)	3.65(3.62–3.68)	4.13(4.06–4.19)	3.17(3.16–3.19)	4.45(4.41–4.49)
Household income per capita(RMB)	24,998.42(23,858.13–26,138.71)	27,096.35(21,241.03–32951.67)	34,563.84(33,781.09–35346.60)	16,642.75(16,139.51–17,145.99)
Household consumption expenditure per capita (RMB)	19,038.72(18,562.12–19,515.33)	20,985.02(18,199.01–23771.02)	22,263.52(21,952.72–22,574.31)	15,243.14(14,641.02–15845.26)
Household healthcare expenditure per capita (RMB)	2081.42(1924.72–2238.12)	3323.66(1365.79–5281.53)	2413.32(2292.75–2533.88)	1961.28(1835.25–2087.31)
Household food expenditure per capita (RMB)	6202.30(6086.71–6317.89)	5156.39(4215.61–6097.16)	8482.94(8404.61–8561.27)	4099.70(4012.29–4187.11)
Weighted				
Urban	0.56(0.55–0.57)	0.64(0.63–0.66)	0.71(0.71–0.72)	0.58(0.57–0.59)
Household family size (person per household)	3.58(3.54–3.61)	4.40(4.33–4.48)	3.20(3.18–3.22)	4.37(4.34–4.41)
Household income per capita (RMB)	24,844.91(23,854.31–25,835.50)	27,743.86(24,930.13–30,557.59)	32,332.23(31,451.31–33,213.15)	18,419.32(17,848.75–18,989.90)
Household consumption expenditure per capita (RMB)	19,671.89(19,007.77–20,336.00)	22,219.44(20,056.04–24382.84)	20,990.20(20,649.93–21,330.46)	16,736.65(16,040.68–17,432.63)
Household healthcare expenditure per capita (RMB)	2095.27(1884.05–2306.48)	2403.83(1379.67–3427.99)	2304.11(2176.63–2431.58)	1881.59(1758.75–2004.44)
Household food expenditure per capita (RMB)	6368.67(6227.95–6509.39)	5260.02(4563.42–5956.62)	8041.95(7950.42–8133.49)	4399.93(4298.04–4501.82)
Sample size	14,017	3745	40,011	9728

*CFPS * China Family Panel Studies, *CGSS*  Chinese General Social Survey, *CHFS*  China Household Finance Survey, *CSS* Chinese Social Survey. Household income per capita, household consumption expenditure per capita, household healthcare expenditure per capita, and household food expenditure per capita were in all comparable prices in 2017 using Consumer Price Index

The Weighted panel shows the results after weighted by cross-sectional weights in each survey. 95% confidence intervals are reported in parentheses

As for household income and expenditures, Table 3 reveals that weighting contributes to enhanced comparability between surveys. Before weighting, CHFS 2017 exhibited the highest levels of household income per capita, household healthcare expenditure per capita, and household food expenditure per capita, with values of 34,563.84 RMB, 22,263.52 RMB, and 8482.94 RMB, respectively. At the same time, CSS 2017 displayed the lowest levels of these variables, with values of 16,642.75 RMB, 15,243.14 RMB, and 4099.70 RMB, respectively. After weighting, CHFS 2017 retained the highest values for household income per capita (32,332.23 RMB) and household food expenditure per capita (8041.95 RMB). Meanwhile, CGSS 2017 exhibited the highest value for household expenditure per capita (22,219.44 RMB). Conversely, CSS 2017 demonstrated the lowest levels of household income per capita (18,419.32 RMB), household expenditure per capita (16,736.65 RMB), and household food expenditure per capita (4399.93 RMB). Before weighting, household healthcare expenditure per capita of CGSS 2017, CHFS 2017, CFPS 2016, and CSS 2017 were 3323.66 RMB, 2413.32 RMB, 2081.42 RMB, and 1961.28 RMB, respectively. While after weighting, they were 2403.83 RMB, 2304.11 RMB, 2095.27 RMB, and 1881.59 RMB, respectively. Hence, while there are disparities in sample selection across multiple surveys, the magnitude of the differences can be reduced after weighting. Therefore, we report the weighted results on the incidence of CHE and MI in the subsequent sections.

### Incidences of CHE and MI during 2016–2017

Table 4 shows the weighted incidences of CHE and MI using different surveys and thresholds in the period of 2016–2017. Despite differences in sampling methods, sample selection, and questionnaire designs across the surveys, the incidences of CHE and MI are mostly consistent under the same threshold. At the 40% nonfood threshold, the incidence of CHE ranged from 14.95% (95% CI: 14.23%-15.68%) to 17.73% (95% CI: 16.90%-18.56%), meaning that approximately one in every six households in China encountered catastrophic health expenditure in 2016–2017. At the 10% threshold, the incidence of CHE ranged from 28.91% (95% CI: 28.30%-29.52%) to 36.18% (95% CI: 35.09%-37.28%). At the 25% threshold, the incidence of CHE ranged from 13.01% (95% CI: 12.56%-13.46%) to 17.42% (95% CI: 16.58%-18.26%).

**Table 4:** The incidence of CHE and MI using different surveys and thresholds, 2016–2017

	CFPS 2016	CGSS 2017	CHFS 2017	CSS 2017
The incidence of CHE (%)				
40% nonfood expenditure	14.95 (14.23–15.68)	15.44 (14.14–16.74)	17.18 (16.67–17.68)	17.73 (16.90–18.56)
10% expenditure	31.70 (30.76–32.64)	31.50 (29.60–33.41)	28.91 (28.30–29.52)	36.18 (35.09–37.28)
25% expenditure	13.50 (12.82–14.19)	14.32 (13.00–15.63)	13.01 (12.56–13.46)	17.42 (16.58–18.26)
The incidence of MI (%)				
1.9 USD poverty line	3.16(2.81–3.51)	4.68(3.87–5.49)	2.01(1.82–2.20)	5.63(5.14–6.13)
3.1 USD poverty line	4.96(4.53–5.39)	4.61(3.82–5.41)	3.65(3.39–3.90)	7.42(6.84–7.99)
Relative poverty line	5.92 (5.45–6.39)	5.22 (4.36–6.07)	5.84 (5.52–6.16)	6.93 (6.37–7.49)
Sample size	14,017	3745	40,011	9728

*CFPS* China Family Panel Studies, *CGSS* Chinese General Social Survey, *CHFS* China Household Finance Survey, *CSS* Chinese Social Survey. 95% confidence intervals are reported in parentheses

Similarly, the incidences of MI across four surveys are mostly consistent under the same threshold. At the 1.9 USD poverty line, the incidence of MI ranged from 2.01% (95% CI: 1.82%-2.20%) to 5.63% (95% CI: 5.14%-6.13%). This implies that approximately one in every twenty households in China fell into poverty due to healthcare expenditures in 2016–2017. At the 3.1 USD poverty line, the incidence of MI ranged from 3.65% (95% CI: 3.39%-3.90%) to 7.42% (95% CI: 6.84%-7.99%). When using the relative poverty line, the incidence of MI ranged from 5.22% (95% CI: 4.36%-6.07%) to 6.93% (95% CI: 6.37%-7.49%). Our results are consistent with previous studies [9, 13].

Table 5 shows the trends in CHE and MI using different thresholds from 2010 to 2020, using CFPS data. During this period, the incidence of CHE displayed a decline over time. Specifically, the incidence of CHE measured by 40% nonfood expenditure threshold decreased from 19.40% (95% CI: 18.52%-20.27%) in 2010 to 9.89% (95% CI: 9.00%-10.77%) in 2020. Similarly, the CHE incidence measured by 10% and 25% threshold experienced a decline from 36.01% (95% CI: 34.94%-37.08%) to 24.56% (95% CI: 23.33%-25.80%), and from 16.41% (95% CI: 15.59%-17.22%) to 9.43% (95% CI: 8.57%-10.29%), respectively. The trends observed in the MI incidence followed a similar pattern, with decreasing from 6.41% (95% CI: 5.88%-6.94%) to 2.77% (95% CI: 2.44%-3.10%) using the 1.9 USD poverty line, from 7.90% (95% CI: 7.31%-8.48%) to 3.97% (95% CI: 3.56%-4.37%) using the 3.1 USD poverty line, and from 7.55% (95% CI: 6.97%-8.13%) to 3.83% (95% CI: 3.37%-4.29%) using the relative poverty line.

**Table 5:** Trends in CHE and MI using different threshold, 2010–2020

	CFPS 2010	CFPS 2012	CFPS 2014	CFPS 2016	CFPS 2018	CFPS 2020
The incidence of CHE (%)						
40% nonfood expenditure	19.40(18.52–20.27)	16.19(15.30–17.07)	14.37(13.59–15.16)	14.95(14.23–15.68)	12.86(12.10–13.63)	9.89(9.00–10.77)
10% expenditure	36.01(34.94–37.08)	31.17(30.06–32.28)	31.50(30.45–32.55)	31.70(30.76–32.64)	29.52(28.44–30.61)	24.56(23.33–25.80)
25% expenditure	16.41(15.59–17.22)	13.04(12.23–13.85)	13.15(12.39–13.90)	13.50(12.82–14.19)	11.92(11.19–12.65)	9.43(8.57–10.29)
The incidence of MI (%)						
1.9 USD poverty line	6.41(5.88–6.94)	3.80(3.35–4.24)	3.45(3.05–3.86)	3.16(2.81–3.51)	2.69(2.36–3.03)	2.77(2.44–3.10)
3.1 USD poverty line	7.90(7.31–8.48)	5.71(5.17–6.25)	5.03(4.55–5.50)	4.96(4.53–5.39)	3.85(3.45–4.25)	3.97(3.56–4.37)
Relative poverty line	7.55(6.97–8.13)	6.52(5.93–7.11)	6.00(5.48–6.51)	5.92(5.45–6.39)	5.20(4.71–5.68)	3.83(3.37–4.29)
Sample size	14,574	12,987	13,789	14,017	14,068	11,401

*CFPS* China Family Panel Studies. 95% confidence intervals are reported in parentheses

### Inequality in financial risk protection

Table 6 shows the weighted incidence of CHE and MI for urban and rural areas in each survey and at each threshold. Table 6 also reports Rural–Urban Gap to represent the absolute difference between urban and rural areas.

**Table 6:** Incidence of CHE and MI, by urban and rural area

	CFPS 2016	CGSS 2017	CHFS 2017	CSS 2017
The incidence of CHE				
40% nonfood expenditure				
Urban (%)	13.07 (12.10–14.03)	10.50 (9.15–11.85)	14.73 (14.16–15.30)	14.77 (13.75–15.78)
Rural (%)	17.67 (16.55–18.79)	24.34 (21.64–27.04)	23.32 (22.27–24.37)	21.83 (20.44–23.21)
Rural–Urban Gap (pp)	4.60***	13.84***	8.59***	7.06***
10% expenditure				
Urban (%)	29.06 (27.76–30.37)	24.50 (22.34–26.66)	26.01 (25.30–26.71)	31.66 (30.27–33.04)
Rural (%)	35.65 (34.25–37.04)	44.07 (40.55–47.60)	36.19 (35.00–37.38)	42.46 (40.71–44.22)
Rural–Urban Gap (pp)	6.59***	19.57***	10.18***	10.80***
25% expenditure				
Urban (%)	11.13 (10.24–12.02)	9.75 (8.37–11.13)	11.08 (10.58–11.59)	14.09 (13.08–15.10)
Rural (%)	16.79 (15.70–17.88)	22.52 (19.83–25.21)	17.84 (16.89–18.79)	22.03 (20.61–23.46)
Rural–Urban Gap (pp)	5.66***	12.77***	6.76***	7.94***
The incidence of MI				
1.9 USD poverty line				
Urban (%)	1.51(1.16–1.86)	2.16(1.43–2.90)	0.76(0.62–0.90)	4.11(3.55–4.66)
Rural (%)	5.43(4.76–6.10)	9.21(7.39–11.03)	5.13(4.57–5.68)	7.78(6.88–8.67)
Rural–Urban Gap (pp)	3.92***	7.05***	4.37***	3.67***
3.1 USD poverty line				
Urban (%)	2.99(2.51–3.48)	2.69(1.97–3.41)	1.80(1.58–2.02)	6.22(5.53–6.92)
Rural (%)	7.62(6.86–8.39)	8.07(6.26–9.87)	8.29(7.60–8.97)	9.10(8.12–10.08)
Rural–Urban Gap (pp)	4.63***	5.38***	6.49***	2.88***
Relative poverty line				
Urban (%)	4.15 (3.59–4.72)	3.50 (2.68–4.33)	4.73 (4.37–5.08)	5.39 (4.74–6.04)
Rural (%)	8.38 (7.57–9.19)	8.29 (6.44–10.15)	8.63 (7.93–9.33)	9.06 (8.08–10.04)
Rural–Urban Gap (pp)	4.23***	4.79***	3.90***	3.67***

*CFPS* China Family Panel Studies, *CGSS*  Chinese General Social Survey, *CHFS* China Household Finance Survey, *CSS* Chinese Social Survey, *pp* represents percentage points. ***, **, * represents statistical significance at 1%, 5%, and 10% levels. 95% confidence intervals are reported in parentheses

All results consistently indicate that the incidence of CHE in urban area is significantly lower than that in rural area, although the magnitude of the differences varies across different surveys and thresholds. At the 40% nonfood threshold, the differences between urban and rural areas, as measured by CFPS, CGSS, CHFS and CSS, were 4.60, 13.84, 8.59, and 7.06 percentage points, respectively. At the 10% threshold, the differences between urban and rural areas, as measured by CFPS, CGSS, CHFS, and CSS, were 6.59, 19.57, 10.18, and 10.80 percentage points, respectively. Similarly, at the 25% threshold, the differences between urban and rural areas in the four surveys were 5.66, 12.77, 6.76, and 7.94 percentage points, respectively.

In terms of urban–rural inequality measured by the incidence of MI, a similar pattern emerges, with urban households consistently experiencing a significantly lower incidence of MI compared to rural residents. At the 1.9 USD poverty line, the differences between urban and rural areas, as measured by CFPS, CGSS, CHFS, and CSS, were 3.92, 7.05, 4.37, and 3.67 percentage points, respectively. At the 3.1 USD poverty line, the differences between urban and rural areas in the four surveys were 4.63, 5.38, 6.49, and 2.88 percentage points, respectively. When using the relative poverty line, the differences between urban and rural areas in the four surveys were 4.23, 4.79, 3.90, and 3.67 percentage points. Moreover, we also conducted a comparison of urban–rural inequality from 2010 to 2020 using CFPS data, as shown in Appendix Table A. The findings align with the results in Table 6, with urban households facing with lower incidence of CHE and MI compared to the rural households in most survey years.

Table 7 shows the weighted incidence of CHE and MI by income quartiles, using various surveys and thresholds. Table 7 also reports Q1-Q4 Gap to show the absolute difference in incidence of CHE and MI between the richest and the poorest income quartiles.

**Table 7:** Incidence of CHE and MI by income quartiles

	CFPS 2016	CGSS 2017	CHFS 2017	CSS 2017
The incidence of CHE				
40% nonfood expenditure				
Q1 (%)	23.21 (21.46–24.95)	25.71 (23.14–28.28)	23.67 (22.51–24.83)	32.49 (30.44–34.54)
Q2 (%)	14.14 (12.76–15.52)	11.03 (8.99–13.07)	17.12 (16.11–18.14)	19.82 (17.94–21.69)
Q3 (%)	12.94 (11.59–14.29)	6.03 (4.21–7.86)	13.95 (13.01–14.88)	13.41 (11.94–14.87)
Q4 (%)	10.16 (8.90–11.41)	2.42 (-0.33–5.16)	14.03 (13.10–14.97)	7.36 (6.23–8.50)
Q1-Q4 Gap (pp)	13.05***	23.29***	9.64***	25.13***
10% expenditure				
Q1 (%)	43.61 (41.57–45.64)	43.75 (40.53–46.97)	35.71 (34.40–37.02)	54.16 (51.89–56.42)
Q2 (%)	31.26 (29.41–33.11)	29.23 (25.89–32.58)	30.01 (28.77–31.25)	41.33 (38.95–43.71)
Q3 (%)	29.27 (27.43–31.12)	15.98 (12.10–19.86)	25.46 (24.29–26.62)	30.82 (28.75–32.89)
Q4 (%)	23.59 (21.82–25.36)	11.84 (6.42–17.26)	24.36 (23.20–25.52)	21.65 (19.75–23.55)
Q1-Q4 Gap (pp)	20.02***	31.91***	11.35***	32.51***
25% expenditure				
Q1 (%)	22.20 (20.50–23.90)	23.79 (21.22–26.35)	17.16 (16.14–18.18)	31.15 (29.09–33.21)
Q2 (%)	13.13 (11.80–14.47)	10.73 (8.62–12.84)	13.21 (12.30–14.12)	20.12 (18.21–22.03)
Q3 (%)	10.51 (9.30–11.72)	4.17 (2.47–5.88)	10.23 (9.41–11.04)	13.62 (12.10–15.13)
Q4 (%)	8.79 (7.61–9.97)	3.02 (0.02–6.02)	11.64 (10.77–12.51)	7.02 (5.90–8.13)
Q1-Q4 Gap (pp)	13.41***	20.77***	5.52***	24.13***
The incidence of MI				
1.9 USD poverty line				
Q1 (%)	8.63(7.48–9.78)	9.85(8.07–11.63)	4.98(4.40–5.56)	11.08(9.72–12.43)
Q2 (%)	2.75(2.11–3.39)	2.00(0.87–3.14)	2.11(1.73–2.49)	8.60(7.26–9.95)
Q3 (%)	1.27(0.81–1.74)	0.25(0.00–0.51)	0.61(0.38–0.84)	3.54(2.75–4.33)
Q4 (%)	0.37(0.15–0.60)	0.00(0.00–0.00)	0.11(0.00–0.23)	1.19(0.72–1.65)
Q1-Q4 Gap (pp)	8.26***	9.85***	4.87***	9.89***
3.1 USD poverty line				
Q1 (%)	11.32(10.02–12.61)	8.65(6.92–10.39)	7.98(7.26–8.71)	10.78(9.38–12.18)
Q2 (%)	5.70(4.79–6.60)	2.77(1.71–3.83)	4.53(3.97–5.09)	11.76(10.26–13.27)
Q3 (%)	2.24(1.67–2.81)	0.75(0.08–1.42)	1.41(1.09–1.74)	6.47(5.38–7.56)
Q4 (%)	1.09(0.68–1.50)	0.00(0.00–0.00)	0.24(0.13–0.36)	2.16(1.52–2.80)
Q1-Q4 Gap (pp)	10.23***	8.65***	7.74***	8.62***
Relative poverty line				
Q1 (%)	9.75 (8.54–10.95)	9.43 (7.58–11.28)	7.72 (7.00–8.45)	11.11 (9.75–12.48)
Q2 (%)	8.05 (6.98–9.12)	3.63 (2.47–4.78)	8.66 (7.88–9.44)	11.29 (9.80–12.79)
Q3 (%)	4.41 (3.60–5.22)	0.74 (0.12–1.36)	4.44 (3.89–5.00)	5.88 (4.82–6.94)
Q4 (%)	1.80 (1.23–2.38)	0.39 (-0.38–1.16)	2.11 (1.73–2.49)	0.77 (0.43–1.11)
Q1-Q4 Gap (pp)	7.95***	9.04***	5.61***	10.34***

*CFPS* China Family Panel Studies, *CGSS* Chinese General Social Survey, *CHFS* China Household Finance Survey, *CSS* Chinese Social Survey, *pp* represents percentage points. Q1, Q2, Q3 and Q4 ranges from the lowest 25% income subgroups to the highest 25% income subgroups

***, **, * represents statistical significance at 1%, 5%, and 10% levels. 95% confidence intervals are reported in parentheses

All results show an inverse relationship between the incidence of CHE and household income, although the magnitude of the differences varies with different surveys, definitions, and thresholds. The disparities between the richest income quartile (Q4) and the poorest income quartile (Q1) were significant across different surveys and thresholds. At the 40% nonfood threshold, the disparities between the richest income quartile and the poorest income quartile for CFPS, CGSS, CHFS, and CSS were 13.05, 23.29, 9.64, and 25.13 percentage points, respectively. At the 10% expenditure threshold, the disparities between the richest and poorest quartiles for each survey were 20.02, 31.91, 11.35, and 32.51 percentage points, respectively. When using 25% expenditure threshold, the disparities between the richest and poorest quartiles for each survey were 13.41, 20.77, 5.52, and 24.13 percentage points, respectively.

The pattern of MI was the same. When using the poverty line of 1.9 US dollars, the disparities between the richest quartile and the poorest quartile were 8.26, 9.85, 4.87, and 9.89 percentage points. When using the poverty line of 3.1 US dollars, the disparities between the richest and poorest quartiles were 10.23, 8.65, 7.74, and 8.62 percentage points. When using the relative poverty line, the disparities between the richest and poorest quartiles were 7.95, 9.04, 5.61, and 10.34 percentage points.

Same to the urban–rural inequality, we also conducted a comparison of income inequality from 2010 to 2020, as is shown in Appendix Table B. In most survey years, the higher-income subgroups experienced lower incidences of CHE and MI compared to the lower-income subgroups, which was in line with the findings of Table 7.

### Influencing factors of CHE and MI

Table 8 shows regression results for the influencing factors of CHE and MI. We used the 40% nonfood definition to measure CHE, and the 1.9 USD poverty line to measure MI. The results indicate that after controlling for other variables, urban residents, higher-income quartiles, and households with larger family sizes were faced with a lower incidence of CHE. The association between household consumption expenditure per capita and CHE was not clearly established. Conversely, higher household food expenditure per capita was associated with a lower incidence of CHE. These patterns were mirrored in the results of MI. After controlling for other variables, urban residents, higher-income subgroups, and households with larger family sizes exhibited a lower incidence of MI. Meanwhile, the household food expenditure per capita was negatively correlated with MI. These results remained robust when using other thresholds (results available upon request). Existing literature also found that residency, income, and household family size were correlated with the incidence of CHE and MI [9, 13, 26].

**Table 8:** Regression results of CHE and MI

	CFPS 2016	CGSS 2017	CHFS 2017	CSS 2017
CHE (40% nonfood expenditure)				
Urban	-0.01	-0.04**	-0.05***	-0.03***
	(0.008)	(0.016)	(0.007)	(0.009)
Q2	-0.07***	-0.08***	-0.03***	-0.12***
	(0.012)	(0.019)	(0.008)	(0.015)
Q3	-0.09***	-0.12***	-0.05***	-0.20***
	(0.012)	(0.023)	(0.008)	(0.015)
Q4	-0.12***	-0.16***	-0.06***	-0.27***
	(0.014)	(0.033)	(0.009)	(0.015)
Household family size	-0.03***	-0.01***	-0.05***	-0.01***
	(0.002)	(0.004)	(0.002)	(0.002)
Household consumption expenditure per capita	0.05***	-0.03***	0.07***	0.04***
	(0.006)	(0.010)	(0.005)	(0.006)
Household food expenditure per capita	-0.07***	-0.02**	-0.11***	-0.02***
	(0.005)	(0.009)	(0.005)	(0.005)
Sample size	13,502	3189	39,499	8299
MI (1.9 USD poverty line)				
Urban	-0.01**	-0.02**	-0.01***	-0.01
	(0.004)	(0.010)	(0.002)	(0.006)
Q2	-0.02***	-0.04***	-0.00**	-0.00
	(0.004)	(0.012)	(0.002)	(0.009)
Q3	-0.02***	-0.06***	-0.01***	-0.04***
	(0.005)	(0.009)	(0.003)	(0.008)
Q4	-0.03***	-^¶^	-0.01***	-0.07***
	(0.005)		(0.003)	(0.008)
Household family size	-0.00***	-0.00	-0.00***	0.00
	(0.001)	(0.002)	(0.001)	(0.001)
Household consumption expenditure per capita	0.00	0.01*	0.00	-0.01***
	(0.002)	(0.005)	(0.001)	(0.002)
Household food expenditure per capita	-0.04***	-0.03***	-0.03***	-0.01***
	(0.003)	(0.005)	(0.002)	(0.003)
Sample size	13,272	2,923	39,513	8,240

^***^, **, * represents statistical significance at 1%, 5%, and 10% levels

*CFPS* China Family Panel Studies, *CGSS* Chinese General Social Survey, *CHFS* China Household Finance Survey, *CSS* Chinese Social Survey

We used Probit regression model, and used cross-sectional weights to weight the samples. Marginal effect was shown in the table, and robust standard error was shown in the parentheses. Q1, Q2, Q3 and Q4 corresponds to income quartiles from the lowest income quartile to the highest income quartile. Rural and Q1 were used as the reference group. All regression controlled the provincial fixed effect. Household consumption expenditure per capita and household food expenditure per capita were taken logarithms before running the regression. ^¶^ indicates that the incidence of MI of the highest income quartile was 0 when using CGSS 2017

## Discussion

Our results suggest that financial risk protection in China’s health system was unsatisfactory compared to other countries, though China has experienced improvements in recent years. According to the WHO [3], when using the 10% expenditure threshold, the average incidence of CHE in the world was 13.2% in 2017. The incidences for the upper middle-income group, Asia countries, and the Western Pacific Region were 16.7%, 16.6%, and 20.2%, respectively. When comparing China with the countries sharing the similar GDP per capita, we found disproportionately high incidences of CHE and MI in China. 7.7% of Russian households and 1.5% of Malaysian households suffered from CHE in the 10% threshold, while our results showed that China’s incidence of CHE was 28.91%-36.18% during 2016–2017. When using the 25% expenditure threshold, the incidence of CHE in the world was 3.8% in 2017, with 5.0% in the upper middle-income group, 5.4% in Asia, and 6.4% in Western Pacific Region. In Russia and Malaysia, the corresponding number was 0.9% and 0.1%, much less than the incidence in China (13.01%-17.42%). The situation of MI is similar. When using the poverty line of 1.9 US dollars, the incidence of MI in the world was 0.9% in 2017, with 1.0% in the upper middle-income group, 1.1% in Asia, and 1.3% in Western Pacific Region. The households in Russian Federation and Malaysia hardly suffered MI, with the incidence of MI close to 0. However, our results showed China’s incidence of MI was 2.01%-5.63% during 2016–2017. Therefore, no matter which survey or threshold was used, the performance of financial risk protection in China’s health system was unsatisfactory when compared internationally.

The poor performance in financial protection in China was largely the consequence of China’s inefficient healthcare delivery system. China had an inefficient and wasteful healthcare delivery system in which public hospitals played a dominant role and primary care facilities had weak capacity [27]. Moreover, public hospitals were incentivized to over-prescribe expensive drugs and tests, leading to rising health expenditures [28]. Although the expansion of health insurance coverage and increasing fiscal investments in health reduced the share of out-of-pocket healthcare expenses in total health expenditures in China, the real financial burden faced by the Chinese population did change much [29].

The social health insurance schemes in China did not provide sufficient financial protection for the Chinese households, especially the low-income households in rural areas. Our results indicate that rural residents and lower-income subgroups are faced with higher incidences of CHE and MI, regardless of surveys and thresholds. In China, there are three primary basic medical insurance – NCMS for rural residents, UEBMI for employed urban residents, and URBMI for child, elderly, disabled, and unemployed residents [10]. Insurance premiums and benefits vary drastically by insurance schemes. For example, the premium for the UEBMI was approximately 4,190 RMB per person, while those for URBMI and NCMS were 780 RMB and 660 RMB in 2018, respectively [9]. Moreover, the reimbursement rate is different for urban and rural residents. Existing literature found that the inpatient reimbursement rate was 44.25% for urban residents, while just 34.58% for rural residents [13]. In addition, reimbursements from social health insurance funds are capped and the number of medical assistance beneficiaries is relatively small in China, meaning that households have to afford a larger share of total health expenditures when they suffer serious illness. Therefore, many Chinese residents still complain about high medical expenses and heavy medical burdens, which are still regarded as a significant social issue [24].

Our study has several limitations. First, due to data availability, we cannot update the data to the latest year. The levels and characteristics of financial risk protection in China’s health system could have changed due to recent health system reforms such as the consolidation of urban and rural resident insurance schemes, payment reform, central volume-based procurement and national drug price negotiation, and the Targeted Poverty Alleviation Campaign in health [30–33]. Second, we could not take more variables into the regression model to further investigate the characteristics of the financial risk protection in China. Further research could explore the relationship between other household characteristics and financial risk protection. Third, we did not discuss all causes for the poor performance in financial protection in China. All these issues should be addressed in future research.

## Conclusions

Based on multiple national household surveys, this study describes the levels and characteristics of financial risk protection in China’s health system using various definitions and thresholds. Our analysis shows that the incidence of CHE and MI are consistent between different surveys, despite varying in sampling method, sample selection, and questionnaire design. When using the 40% nonfood threshold, the incidence of CHE varied from 14.95% to 17.73% by survey during the period of 2016–2017. When using the poverty line of 1.9 US dollars, the incidence of MI varied from 2.01% to 5.63% during the same period. Moreover, our results suggest there were large variations by urban/rural residency and across income groups. Providing better financial protection for low-income groups in rural areas is the key to improve financial protection in China.

### Supplementary Information

**Additional file 1.**

## Data Availability

We used four nationally representative household surveys in China: China Family Panel Studies (CFPS), Chinese General Social Survey (CGSS), China Household Finance Survey (CHFS), and Chinese Social Survey (CSS). They are all publicly available. CFPS data files are available from http://www.isss.pku.edu.cn/cfps/. CGSS data files are available from http://cgss.ruc.edu.cn/. CHFS data files are available from https://chfs.swufe.edu.cn/. CSS data files are available from http://csqr.cass.cn/.

## Abbreviations

CHE: Catastrophic health expenditure
MI: Medical impoverishment
UHC: Universal health coverage
SDG: Sustainable Development Goals
WHO: World Health Organization
WB: World Bank
NCMS: New Cooperative Medical Scheme
UEBMI: Urban Employee Basic Medical Insurance
URBMI: Urban Resident Basic Medical Insurance
CFPS: China Family Panel Studies
CGSS: Chinese General Social Survey
CHFS: China Household Finance Survey
CSS: Chinese Social Survey
USD: US dollars
PPP: Purchasing Power Parity
CPI: Consumer Price Index
CI: Confidence interval
pp: Percentage point
