# Supplementary material for: Understanding financial risk protection in China’s health system: a descriptive analysis using data from multiple national household surveys
Source: BMC Public Health. 2023 Sep 19;23:1820. doi: 10.1186/s12889-023-16679-4 (PMC10508013; doi:10.1186/s12889-023-16679-4)
Supplement: Supplementary file 1 — Additional file 1. [file 12889_2023_16679_MOESM1_ESM.docx]

**Appendix**

**Table A. Trends in CHE and MI by urban and rural area，2010-2020.**

|  | **CFPS 2010** | **CFPS 2012** | **CFPS 2014** | **CFPS 2016** | **CFPS 2018** | **CFPS 2020** |
| --- | --- | --- | --- | --- | --- | --- |
| **The incidence of CHE** |  |  |  |  |  |  |
| **40% nonfood expenditure** |  |  |  |  |  |  |
| Urban (%) | 16.49(15.29-17.68) | 13.97(12.72-15.21) | 11.84(10.80-12.88) | 13.07 (12.10-14.03) | 10.26(9.28-11.25) | 8.92(7.65-10.20) |
| Rural (%) | 22.19(20.92-23.45) | 18.56(17.30-19.83) | 17.34(16.14-18.54) | 17.67 (16.55-18.79) | 17.58(16.36-18.81) | 11.99(10.71-13.27) |
| Rural-Urban Gap (pp) | 5.70*** | 4.59*** | 5.50*** | 4.60*** | 7.32*** | 3.07*** |
| **10% expenditure** |  |  |  |  |  |  |
| Urban (%) | 33.35(31.82-34.89) | 29.09(27.48-30.71) | 26.89(25.45-28.33) | 29.06 (27.76-30.37) | 25.48(24.00-26.96) | 22.04(20.39-23.69) |
| Rural (%) | 38.55(37.07-40.03) | 33.41(31.89-34.93) | 36.83(35.30-38.36) | 35.65 (34.25-37.04) | 37.04(35.50-38.58) | 28.90(27.14-30.66) |
| Rural-Urban Gap (pp) | 5.20*** | 4.32*** | 9.94*** | 6.59*** | 11.56*** | 6.86*** |
| **25% expenditure** |  |  |  |  |  |  |
| Urban (%) | 13.83(12.73-14.94) | 11.68(10.53-12.84) | 10.25(9.27-11.23) | 11.13 (10.24-12.02) | 9.07(8.15-9.99) | 8.23(6.98-9.47) |
| Rural (%) | 18.87(17.68-20.06) | 14.46(13.32-15.60) | 16.48(15.31-17.65) | 16.79 (15.70-17.88) | 16.97(15.77-18.18) | 11.79(10.57-13.01) |
| Rural-Urban Gap (pp) | 5.04*** | 2.78*** | 6.23*** | 5.66*** | 7.90*** | 3.56*** |
| **The incidence of MI** |  |  |  |  |  |  |
| **1.9 USD poverty line** |  |  |  |  |  |  |
| Urban (%) | 3.59(3.02-4.16) | 2.11(1.60-2.62) | 1.52(1.12-1.92) | 1.51(1.16-1.86) | 1.39(1.02-1.75) | 1.23(0.90-1.56) |
| Rural (%) | 9.11(8.24-9.97) | 5.63(4.88-6.37) | 5.63(4.90-6.36) | 5.43(4.76-6.10) | 4.90(4.25-5.56) | 5.39(4.70-6.08) |
| Rural-Urban Gap (pp) | 5.52*** | 3.52*** | 4.11*** | 3.92*** | 3.51*** | 4.16*** |
| **3.1 USD poverty line** |  |  |  |  |  |  |
| Urban (%) | 5.89(5.14-6.63) | 3.54(2.88-4.20) | 2.96(2.42-3.50) | 2.99(2.51-3.48) | 1.97(1.56-2.39) | 2.15(1.73-2.58) |
| Rural (%) | 9.82(8.93-10.71) | 8.04(7.17-8.91) | 7.39(6.58-8.20) | 7.62(6.86-8.39) | 7.09(6.29-7.90) | 7.10(6.30-7.91) |
| Rural-Urban Gap (pp) | 3.93*** | 4.50*** | 4.43*** | 4.63*** | 5.12*** | 4.95*** |
| **Relative poverty line** |  |  |  |  |  |  |
| Urban (%) | 5.12(4.41-5.83) | 4.61(3.86-5.36) | 4.38(3.71-5.05) | 4.15 (3.59-4.72) | 3.49(2.91-4.08) | 2.76(2.21-3.32) |
| Rural (%) | 9.87(8.97-10.77) | 8.63(7.70-9.55) | 7.83(7.01-8.64) | 8.38 (7.57-9.19) | 8.19(7.35-9.04) | 5.77(4.91-6.62) |
| Rural-Urban Gap (pp) | 4.75*** | 4.02*** | 3.45*** | 4.23*** | 4.70*** | 3.01*** |

Note: CFPS = China Family Panel Studies. pp represents percentage points. ***, **, * represents statistical significance at 1%, 5%, and 10% levels. 95% confidence intervals are reported in parentheses.

**Table B. Trends in CHE and MI by income quartiles, 2010-2020.**

|  | **CFPS 2010** | **CFPS 2012** | **CFPS 2014** | **CFPS 2016** | **CFPS 2018** | **CFPS 2020** |
| --- | --- | --- | --- | --- | --- | --- |
| **The incidence of CHE** |  |  |  |  |  |  |
| **40% nonfood expenditure** |  |  |  |  |  |  |
| Q1 (%) | 29.73(27.69-31.77) | 22.77(20.78-24.77) | 20.69(18.90-22.49) | 23.21(21.46-24.95) | 23.75(21.77-25.73) | 16.90(14.88-18.92) |
| Q2 (%) | 19.52(17.81-21.23) | 16.19(14.42-17.95) | 15.16(13.45-16.86) | 14.14(12.76-15.52) | 12.61(11.22-14.00) | 9.97(8.46-11.49) |
| Q3 (%) | 14.77(13.25-16.28) | 14.76(13.02-16.50) | 11.05(9.64-12.45) | 12.94(11.59-14.29) | 10.00(8.58-11.42) | 9.18(6.88-11.49) |
| Q4 (%) | 13.97(12.35-15.60) | 11.04(9.55-12.53) | 10.19(8.76-11.62) | 10.16(8.90-11.41) | 7.85(6.55-9.14) | 4.99(3.87-6.11) |
| Q1-Q4 Gap (pp) | 15.76*** | 11.73*** | 10.50*** | 13.05*** | 15.90*** | 11.91*** |
| **10% expenditure** |  |  |  |  |  |  |
| Q1 (%) | 45.86(43.63-48.08) | 36.63(34.37-38.89) | 40.98(38.79-43.17) | 43.61(41.57-45.64) | 43.19(40.93-45.44) | 33.88(31.43-36.33) |
| Q2 (%) | 37.11(35.03-39.20) | 33.47(31.24-35.70) | 35.01(32.77-37.26) | 31.26(29.41-33.11) | 32.37(30.31-34.43) | 26.84(24.60-29.08) |
| Q3 (%) | 31.63(29.62-33.64) | 29.56(27.37-31.75) | 27.21(25.19-29.23) | 29.27(27.43-31.12) | 25.88(23.82-27.94) | 22.28(19.53-25.04) |
| Q4 (%) | 29.70(27.54-31.85) | 25.03(22.89-27.17) | 22.27(20.29-24.25) | 23.59(21.82-25.36) | 20.69(18.54-22.84) | 17.42(15.05-19.79) |
| Q1-Q4 Gap (pp) | 16.16*** | 11.60*** | 18.71*** | 20.02*** | 22.50*** | 16.46*** |
| **25% expenditure** |  |  |  |  |  |  |
| Q1 (%) | 25.13(23.20-27.06) | 17.80(15.97-19.63) | 19.61(17.84-21.37) | 22.20(20.50-23.90) | 21.90(19.98-23.81) | 16.49(14.56-18.42) |
| Q2 (%) | 16.51(14.92-18.10) | 13.34(11.71-14.97) | 14.15(12.52-15.78) | 13.13(11.80-14.47) | 11.89(10.54-13.25) | 9.16(7.72-10.61) |
| Q3 (%) | 12.71(11.28-14.14) | 11.55(9.99-13.10) | 9.95(8.61-11.29) | 10.51(9.30-11.72) | 9.34(8.00-10.68) | 8.24(5.99-10.49) |
| Q4 (%) | 11.57(10.08-13.06) | 9.47(8.07-10.87) | 8.50(7.18-9.82) | 8.79(7.61-9.97) | 7.10(5.89-8.31) | 5.22(4.06-6.37) |
| Q1-Q4 Gap (pp) | 13.56*** | 8.33*** | 11.11*** | 13.41*** | 14.80*** | 11.27*** |
| **The incidence of MI** |  |  |  |  |  |  |
| **1.9 USD poverty line** |  |  |  |  |  |  |
| Q1 (%) | 12.40(10.95-13.85) | 7.15(5.96-8.33) | 7.61(6.45-8.77) | 8.63(7.48-9.78) | 8.11(6.88-9.33) | 8.16(6.95-9.37) |
| Q2 (%) | 7.87(6.75-8.99) | 3.53(2.67-4.39) | 3.33(2.49-4.18) | 2.75(2.11-3.39) | 2.86(2.15-3.57) | 3.23(2.49-3.96) |
| Q3 (%) | 4.18(3.32-5.04) | 3.35(2.49-4.21) | 1.54(0.98-2.11) | 1.27(0.81-1.74) | 0.83(0.48-1.17) | 0.81(0.47-1.16) |
| Q4 (%) | 1.21(0.74-1.67) | 1.17(0.67-1.67) | 0.53(0.19-0.87) | 0.37(0.15-0.60) | 0.34(0.10-0.58) | 0.30(0.09-0.51) |
| Q1-Q4 Gap (pp) | 11.19*** | 5.98*** | 7.08*** | 8.26*** | 7.77*** | 7.86*** |
| **3.1 USD poverty line** |  |  |  |  |  |  |
| Q1 (%) | 10.51(9.19-11.82) | 9.14(7.79-10.49) | 9.27(7.99-10.56) | 11.32(10.02-12.61) | 10.12(8.75-11.48) | 10.22(8.84-11.60) |
| Q2 (%) | 10.85(9.53-12.18) | 6.30(5.19-7.40) | 6.48(5.37-7.60) | 5.70(4.79-6.60) | 5.02(4.11-5.92) | 5.35(4.41-6.28) |
| Q3 (%) | 6.64(5.57-7.71) | 4.77(3.79-5.75) | 2.72(2.03-3.41) | 2.24(1.67-2.81) | 1.56(1.04-2.08) | 1.68(1.22-2.15) |
| Q4 (%) | 3.41(2.54-4.28) | 2.63(1.85-3.40) | 1.23(0.77-1.68) | 1.09(0.68-1.50) | 0.47(0.20-0.74) | 0.40(0.14-0.67) |
| Q1-Q4 Gap (pp) | 7.10*** | 6.51*** | 8.04*** | 10.23*** | 9.65*** | 9.82*** |
| **Relative poverty line** |  |  |  |  |  |  |
| Q1 (%) | 11.66(10.26-13.05) | 8.76(7.40-10.12) | 10.17(8.85-11.48) | 9.75(8.54-10.95) | 9.18(7.87-10.49) | 6.50(5.30-7.70) |
| Q2 (%) | 10.70(9.36-12.05) | 8.86(7.52-10.21) | 8.27(6.99-9.56) | 8.05(6.98-9.12) | 7.48(6.40-8.56) | 6.25(5.08-7.42) |
| Q3 (%) | 5.75(4.74-6.76) | 5.51(4.43-6.60) | 3.27(2.53-4.00) | 4.41(3.60-5.22) | 4.58(3.55-5.61) | 2.52(1.69-3.35) |
| Q4 (%) | 1.93(1.29-2.56) | 2.94(2.11-3.76) | 2.00(1.37-2.64) | 1.80(1.23-2.38) | 1.04(0.64-1.44) | 0.89(0.49-1.28) |
| Q1-Q4 Gap (pp) | 9.73*** | 5.82*** | 8.17*** | 7.95*** | 8.14*** | 5.61*** |

Note: Note: CFPS = China Family Panel Studies. pp represents percentage points. Q1, Q2, Q3 and Q4 ranges from the lowest 25% income subgroups to the highest 25% income subgroups. ***, **, * represents statistical significance at 1%, 5%, and 10% levels. 95% confidence intervals are reported in parentheses.
